# Reciprocal effects of conditioned medium on gene and protein expression of limbal epithelial cells and limbal fibroblasts in congenital aniridia

**DOI:** 10.1371/journal.pone.0327167

**Published:** 2025-07-07

**Authors:** Maximilian Berger, Nóra Szentmáry, Tim Berger, Julia Zimmermann, Simon Trusen, Berthold Seitz, Fabian N. Fries, Shweta Suiwal, Maryam Amini, Tanja Stachon

**Affiliations:** 1 Dr. Rolf M. Schwiete Center for Limbal Stem Cell and Congenital Aniridia Research, Saarland University, Homburg/Saar, Germany; 2 Department of Ophthalmology, Saarland University Medical Center, Homburg/Saar, Germany; Institute of Science Tokyo, JAPAN

## Abstract

Congenital aniridia is marked by substantial inflammatory changes to the ocular surface. However the exact mechanisms of epithelial-stromal interaction are not fully understood. The purpose of this study was to investigate inflammatory cytokine expression in limbal epithelial cells and fibroblasts following exposure to each other’s conditioned medium (CM). Healthy primary limbal epithelial cells (pLECs) and healthy (LFC) or aniridia primary limbal fibroblasts (AN-LFC) were isolated. A PAX6-deficient limbal epithelial cell line (mut-LSCs) modeled aniridia. pLECs underwent siRNA-mediated PAX6 knockdown (siPAX6 pLECs) with control cells transfected with non-specific siRNA (siCtrl pLECs). siCtrl and siPAX6 pLECs were treated with LFC-CM and AN-LFC-CM for 24 hours, while LFC and AN-LFC were treated with pLECs-CM and mut-LSCs-CM for 48 hours. Gene and protein expression of IL-1β, IL-6, IL-8, TNF-α and VEGF-A were measured using qPCR and ELISA. Except for an increased IL-8 protein expression in siPAX6 pLECs treated with LFC-CM, gene and protein levels of inflammatory biomarkers remained unchanged in siCtrl and siPAX6 pLECs, regardless of treatment with LFC-CM or AN-LFC-CM. In LFCs, pLECs-CM decreased TNF-α mRNA and IL-8 protein, while increasing IL-1β, IL-6, IL-8 mRNA and IL-6 protein. LFCs treated with mut-LSCs-CM showed decreased TNF-α mRNA and increased IL-6 protein. AN-LFCs treated with pLECs-CM showed increased IL-6, IL-8 and VEGF-A mRNA and IL-6 protein. mut-LSCs-CM did not alter AN-LFC expression. Limbal fibroblasts’ secretome minimally inflames limbal epithelial cells, suggesting a supportive niche role. In contrast, pLECs-CM induces a stronger fibroblast response, indicating abnormal interactions in congenital aniridia.

## Introduction

Congenital aniridia is a bilateral, panocular disorder with an estimated incidence of 1:50,000–1:100,000 live births and is primarily due to haploinsufficiency of the paired box 6 (PAX6) gene, which results from an autosomal dominant mutation on chromosome 11 [1]. This mutation is distinguished by a high penetrance and a variable phenotypic expression [2]. The transcription factor PAX6 plays a pivotal role in regulating ocular development and maintaining the functionality of both the anterior and posterior segments of the eye throughout life, with sustained PAX6 expression in the limbal and corneal epithelium, the ciliary body and the ganglion cell layer of the retina [3]. The most notable feature of congenital aniridia is iris hypoplasia, which may manifest as a partial to complete loss of the iris tissue. Visual acuity is impaired due to foveal hypoplasia, the onset of secondary glaucoma, juvenile cataract and aniridia-associated keratopathy (AAK) [4]. AAK is characterized by limbal stem cell deficiency, a pathological state caused by the progressive loss of functional limbal epithelial stem cells (LESC), leading to conjunctivalization of the cornea and chronic ocular surface inflammation [5,6]. A patient-centered approach to treatment is essential, as surgical interventions, particularly those involving manipulation of the limbus, have the potential to exacerbate AAK [7,8]. Under physiological conditions, the continuous renewal of the mature corneal epithelium is ensured by the survival, proliferation and differentiation of the LESC in their stem cell niche [9]. LESC are presumably distributed in clusters within the basal epithelial layers of the crypts situated between the vascularized Vogt´s palisades (S1 Fig) [10–12]. The stem cell phenotype is partially conserved through interaction with specialized niche cells, namely those present in the limbal stroma [9]. Limbal stromal cells extend through interruptions in the basement membrane to project onto basal epithelial cells, which exhibit morphological stem cell features and appear to promote the undifferentiated state of LESC [12]. However, the precise mechanisms of epithelial-stromal interaction and the manner by which the limbal stroma modulates the stem cell properties of LESC remains to be fully elucidated.

The maintenance of corneal transparency is dependent on a distinctive immune status that inhibits inadequate, excessive local inflammatory responses [13]. In congenital aniridia, PAX6 haploinsufficiency disrupts the physiological function of limbal epithelial cells, potentially impairing their interaction with limbal fibroblasts, which serve as their niche cells [6,9,14,15]. This compromises the integrity of the limbal stem cell niche, leading to ocular surface inflammation, formation of an opaque pannus tissue and recurrent epithelial defects, ultimately resulting in severe visual loss [16]. Inflammatory changes to the ocular surface include the infiltration of the limbal epithelium with CD18-positive immune cells from the corneal stroma, labeling of the subepithelial pannus tissue with CD68, a macrophage marker, and an increase in various proinflammatory cytokines such as interleukins (IL)-1β, IL-9 and IL-17A in the tear fluid [15,17,18]. The inflammatory state and damage to the ocular surface appear to worsen as AAK advances, thereby creating a potential vicious circle [3]. Nevertheless, in late AAK stages, ocular surface inflammation already seems to be less prominent [19].

The purpose of this study was to investigate the gene and protein expression of the key inflammatory cytokines IL-1β, IL-6, IL-8, tumor necrosis factor-α (TNF-α) and the angiogenic molecule vascular endothelial growth factor A (VEGF-A) in limbal epithelial cells and limbal fibroblasts in an indirect co-culture system to better understand the reciprocal interactions of these cell compartments and the pathophysiology of AAK.

## Materials and methods

This study was conducted in accordance to the tenets of the Declaration of Helsinki and the use of human tissue was approved by the Ethics Committee of Saarland/Germany (no. 178/22). All donors have provided written consent for the utilization of their biological samples for research purposes. Descriptive data of donors are represented in S1 Table. The archived samples were accessed for research purposes only from December 2022 to July 2023. The authors accessed anonymized samples with no identifiable donor information at any stage of the study. An illustration of the experimental workflow is presented in S2 Fig, which was created using the BioRender Software (Toronto, Canada).

### Cell culture

Cell cultivation was performed in an incubator (Heracell 240i, Thermo Fisher Scientific, Waltham, MA, USA) at 37°C under an atmosphere of 95% relative humidity and 5% CO_2_. Cell culture experiments were conducted within a biological safety cabinet class II (Safe 2020, Thermo Fisher Scientific) and aseptic techniques were employed.

### Isolation and culture of primary human limbal epithelial cells

Healthy primary limbal epithelial cells (pLECs) were isolated from human corneoscleral buttons, as previously described by Latta et al., using a 1.5 mm disposable biopsy punch (KAI Europe GmbH, Solingen, Germany) [20]. Tissue specimens were placed in one 24-well (Sarstedt AG & Co. KG, Nümbrecht, Germany) containing 100 µl of collagenase A (4 mg/ml, Roche Diagnostics GmbH, Mannheim, Germany) and 700 µl of keratinocyte serum-free medium (KSFM, Thermo Fisher Scientific) supplemented with bovine pituitary extract (BPE, 0.05 mg/ml, Thermo Fisher Scientific), human recombinant epidermal growth factor (EGF, 0.005 µg/ml, Thermo Fisher Scientific) and 1% (v/v) of penicillin and streptomycin (P/S, Sigma-Aldrich, St. Louis, MO, USA) for overnight enzymatic digestion. The cell suspension was filtered through a 20 µm pore-sized cell strainer (Bel-Art, Wayne, NJ, USA), which was then inverted over one 6-well (Sarstedt AG & Co. KG) and washed with 2.5 ml of 0.05% trypsin-ethylenediaminetetraacetic acid (EDTA) solution (Sigma-Aldrich) to detach the clustered epithelial cells. Trypsinization was neutralized by the addition of 2.5 ml of Dulbecco´s Modified Eagle Medium (DMEM, Thermo Fisher Scientific) + 5% fetal calf serum (FCS, Thermo Fisher Scientific) + 1% P/S. The cell suspension was centrifuged (Megafuge 16R, Thermo Fisher Scientific) at 200 g for 5 min, after which the cell pellet was resuspended in 500 µl of KSFM + BPE/EGF + 1% P/S and transferred to one 24-well. Upon reaching 80% confluency, pLECs were subcultured, as previously described, initially into two 12-wells (Sarstedt AG & Co. KG) with 1.5 ml of KSFM + BPE/EGF + 1% P/S, followed by passage into four 12-wells and lastly into four 6-wells with 2.5 ml of KSFM + BPE/EGF + 1% P/S. Medium was changed every two to three days.

### PAX6 deficient limbal epithelial cell line and siRNA-mediated PAX6 knockdown

Due to limited availability of limbal epithelial cells from donors with congenital aniridia, a telomerase-immortalized epithelial cell line containing a nonsense mutation in the PAX6 gene (PAX6+/- T-LSCs, subsequently referred to as mut-LSCs), introduced through CRISPR/Cas9 technology, kindly provided by the research group of Daniel Aberdam, was employed as a surrogate for obtaining the aniridia epithelial cell line conditioned medium (mut-LSCs-CM) [21]. The mut-LSCs were seeded at a density of 4,200 cells/cm2 and cultured in 6-wells with KSFM + BPE/EGF + calcium chloride (0.4 mM) + glutamine (2 mM) + 1% P/S, according to the protocol established by Roux et al. [21]. Medium was changed every two to three days.

For treatment purposes, pLECs were additionally used to generate a small interfering ribonucleic acid (siRNA)-based model for PAX6 knockdown, as described by Latta et al. [22]. At 70% confluency the pLECs were transfected in 6-wells with either 5 nM siRNA targeting PAX6 (siPAX6 pLECs, MWG Eurofins, Luxembourg) or 5 nM non-specific control siRNA (siCtrl pLECs, MWG Eurofins) and 5 µl Lipofectamine dissolved in 150 µl Opti-MEM + GlutaMAX (Gibco, Carlsbad, CA, USA). The incubation period was 48 hours prior to treatment with conditioned medium (CM). While mut-LSCs and siPAX6 pLECs serve as valuable substitutes for primary limbal epithelial cells from patients with congenital aniridia, they have inherent limitations [22,23].

### Isolation and culture of primary human limbal fibroblasts

Primary human limbal fibroblasts were isolated from biopsies obtained from healthy donors prior to corneal harvesting (LFC) and from patients with AAK (AN-LFC) during surgery, as previously described by Li et al. [24]. The tissue samples were incubated overnight at 37°C in a mixture of 100 µl of collagenase A (4 mg/ml) and 700 µl of KSFM + BPE/EGF + 1% P/S. The cell suspension was filtered through a 40 µm pore-sized cell strainer (Bel-Art), followed by centrifugation at 1500 rpm for 5 min. The cell pellet, comprising the limbal fibroblast fraction, was resuspended in DMEM + 5% FCS + 1% P/S and seeded into one 6-well with a total volume of 2.5 ml of DMEM + 5% FCS + 1% P/S. Limbal fibroblasts were subcultured at 90% confluency by adding a 0.05% trypsin-EDTA solution (1.5 ml for one 6-well, 4 ml for one 75-cm2 culture flask) for 7 min and trypsinization was neutralized with equal volumes of DMEM + 5% FCS. The detached cells were centrifuged at 1500 rpm for 5 min, initially seeded into one 75-cm2 culture flask with 10 ml of DMEM + 5% FCS + 1% P/S and subsequently split into six 75-cm2 culture flasks (Sarstedt AG & Co. KG). Medium was changed every two to three days.

### Conditioned medium

The cell culture supernatants were collected exclusively from pLECs and mut-LSCs in 6-wells and from LFC and AN-LFC in 75-cm^2^ culture flasks after 48 hours of incubation, targeting a confluency of 60–80%. During the conditioning phase, calcium chloride and glutamine were not provided as additional supplements for the mut-LSCs. The CM were centrifuged at 3000 g for 5 min to remove cellular debris, sterile filtered through a 0.22 µm syringe filter (Sarstedt AG & Co. KG) and stored temporarily at −80°C. The corresponding CM were thawed at room temperature and pooled. To compensate for the accelerated growth behavior of the mut-LSCs, the protein concentration of the mut-LSCs-CM was adjusted to the CM of the untreated primary limbal epithelial cells (pLECs-CM) with KSFM + BPE/EGF + 1% P/S. Therefore, the protein concentration of the pLECs-CM and mut-LSCs-CM was quantified using a Pierce BCA Protein Assay Kit (Thermo Fisher Scientific), according to the manufacturer’s instructions. A protein standard curve was generated using a dilution series of bovine serum albumin (Sigma-Aldrich). The intensity of the colorimetric reaction was determined at a wavelength of 562 nm with an absorption microplate reader (Infinite F50, Tecan Group AG, Männedorf, Switzerland). The CM were stored at −80°C.

### Treatment of limbal epithelial cells and limbal fibroblasts

Prior to treatment, limbal epithelial cells and limbal fibroblasts were rinsed once with phosphate buffered saline (Sigma-Aldrich). The conditioned media of healthy (LFC-CM) and aniridia primary limbal fibroblasts (AN-LFC-CM) were diluted 1:1 with KSFM + BPE/EGF + 1% P/S, while the pLECs-CM and mut-LSCs-CM were diluted 1:1 with DMEM + 5% FCS + 1% P/S. The control medium (Ctrl-M) was prepared in an identical manner for treatment of both cell types and consisted of a 1:1 mixture of the standard growth media KSFM + BPE/EGF + 1% P/S and DMEM + 5% FCS + 1% P/S, without prior cell conditioning. Subsequently, siCtrl pLECs and siPAX6 pLECs were treated in 6-wells for a period of 24 hours with 2.5 ml of either LFC-CM, AN-LFC-CM or Ctrl-M. LFC and AN-LFC were treated in 75-cm^2^ culture flasks for 48 hours with 10 ml of either pLECs-CM, mut-LSCs-CM or Ctrl-M. Following treatment, the cell culture supernatant was centrifuged at 3000 g for 5 min to remove cellular debris and aliquoted into 2 ml tubes for storage at −80°C. Cells were harvested via trypsinization and the cell pellet was immediately stored at −80°C in order to limit RNase activity until further use.

### RNA extraction and cDNA synthesis

Total RNA was extracted from limbal epithelial cells using the RNA/DNA/Protein Purification Plus Kit (Norgen Biotek Corp., Thorold, ON, Canada) and from limbal fibroblasts using the Total RNA Purification Plus Micro Kit (Norgen Biotek Corp.), in accordance with the manufacturer´s instructions. The lysis buffer containing guanidinium salts was additionally supplemented with β-mercaptoethanol (Sigma-Aldrich) to prevent RNA degradation. The RNA concentration and purity were determined spectrophotometrically (ScanDrop 100, Analytik Jena GmbH, Jena, Germany) by measuring absorbance at 260 nm and calculating the A260/A230 and A260/A280 ratios. The RNA was stored at −80°C.

For the synthesis of complementary deoxyribonucleic acid (cDNA), 1 µg of total RNA was reverse transcribed using the OneTaq RT-PCR Kit (New England Biolabs, Ipswich, MA, USA) and the MiniAmp thermal cycler (Thermo Fisher Scientific). The manufacturer´s protocol was adhered to and the cDNA was stored at −20°C.

### Quantitative polymerase chain reaction

The QuantStudio 5 Real-Time PCR System (Thermo Fisher Scientific) was employed for the PCR reaction and quantitative measurement of the obtained amount of DNA. Each qPCR reaction comprised 1 µl of primer (Qiagen N.V., Venlo, The Netherlands), 5 µl of AceQ Universal SYBR qPCR Master Mix (Vazyme Biotech Co., Nanjing, China), 3 µl of nuclease-free water and 1 µl of cDNA. An initial denaturation step at 95°C for 5 min was followed by 40 cycles of denaturation at 95°C for 10 s, primer hybridization at 60°C for 30 s and elongation while heating up to 95°C. The cycle threshold (Ct) values were determined in duplicates and normalized to the arithmetic mean of the reference genes TATA-binding protein (TBP) and β-glucuronidase (GUSB). The relative changes in gene expression were calculated using the 2^-ΔΔCT^ method. The used primers are listed in S2 Table.

### Enzyme-linked immunosorbent assay

An Enzyme-linked immunosorbent assay (ELISA) was chosen as the most appropriate technique for quantifying cytokine secretion due to its high sensitivity and specificity in detecting soluble proteins in complex media. The concentrations of IL-1β, IL-6, IL-8, TNF-α and VEGF-A were quantified in duplicates in the cell culture supernatants of limbal fibroblasts and limbal epithelial cells after treatment, using the respective DuoSet ELISA Kit (R&D Systems Inc., Minneapolis, MN, USA), according to the manufacturer´s instructions. The optical density was evaluated at a wavelength of 450 nm using an absorption microplate reader (Infinite F50, Tecan Group AG) and an eight point standard curve was generated with the provided protein standards. The cytokine concentrations were related to the respective total protein content of the cell lysate (pg cytokine/ mg of total protein) and the quotient was subjected to further statistical analysis. The used ELISA kits are listed in S2 Table.

### Western blot

Total protein was extracted from siCtrl pLECs and siPAX6 pLECs using the RNA/DNA/Protein Purification Plus Kit (Norgen Biotek Corp.) and protein concentration was measured using the Pierce Bradford Protein Assay Kit (Thermo Fisher Scientific), according to the manufacturer’s instructions. Protein samples with 20 µg of total protein were heated in Laemmli sample buffer (Bio-Rad Laboratories, Hercules, CA, USA) for 5 min at 95°C and separated by electrophoresis (Analytik Jena GmbH & Co. KG) at a constant current of 100 V on a NuPAGE 4–12% Bis-Tris precast gel (Thermo Fisher Scientific). Protein transfer onto a nitrocellulose membrane was conducted using the Trans-Blot Turbo System (Bio-Rad Laboratories) and total protein normalization was achieved through the utilization of No-Stain Protein Labeling Reagent (Thermo Fisher Scientific). Total protein normalization was used to ensure equal protein loading across all treatment groups. The membrane was incubated overnight at 4°C with a primary antibody targeting the PAX6 protein (Merck KGaA, Darmstadt, Germany) at a dilution of 1:1,000 in WesternFroxx anti-rabbit HRP solution (neoFroxx GmbH, Einhausen, Germany). Chemiluminescence was detected using the iBright Imaging System (Thermo Fisher Scientific) and protein bands were normalized to the respective total protein content.

### Statistical analysis

GraphPad Prism 10.1.2 Software (GraphPad Software, Boston, MA, USA) was used for the data analysis and graphical display of Figs 2–5. Mean and standard deviation (SD) were reported for the generated S3–S6 Tables. Statistical analysis was conducted using a two-way ANOVA followed by Dunnett´s multiple comparisons test, with p-values below 0.05 considered statistically significant.

**Fig 1:**
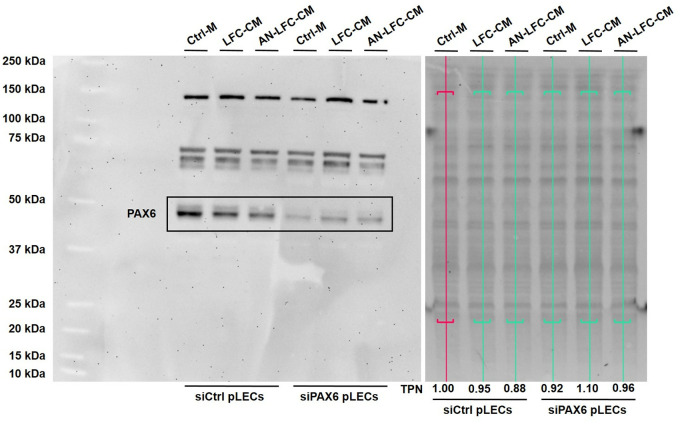
Representative western blot of successful siRNA-mediated knockdown of PAX6 protein (molecular weight of 48 kDa) in primary limbal epithelial cells transfected with siRNA targeting PAX6 (siPAX6 pLECs) in contrast to primary limbal epithelial cells transfected with a non-specific control siRNA (siCtrl pLECs). The loaded protein amount between the treatment groups with control medium (Ctrl-M), conditioned medium of healthy limbal fibroblasts (LFC-CM) and aniridia limbal fibroblasts (AN-LFC-CM) was assessed by total protein normalization (TPN).

**Fig 2:**
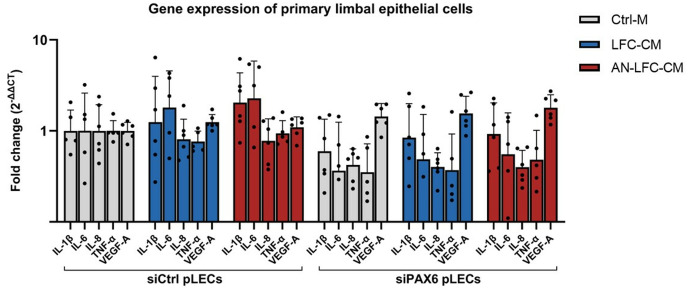
mRNA level of interleukins (IL-1β, IL-6, IL-8), tumor necrosis factor-α (TNF-α) and vascular endothelial growth factor A (VEGF-A) in primary limbal epithelial cells transfected with a non-specific control siRNA (siCtrl pLECs) and limbal epithelial cells transfected with siRNA targeting PAX6 (siPAX6 pLECs) after treatment with conditioned medium from healthy limbal fibroblasts (LFC-CM) or conditioned medium from aniridia limbal fibroblasts (AN-LFC-CM). Fold changes are expressed in relation to the siCtrl pLECs with control medium (Ctrl-M) as geometric mean ± geometric standard deviation.

**Fig 3:**
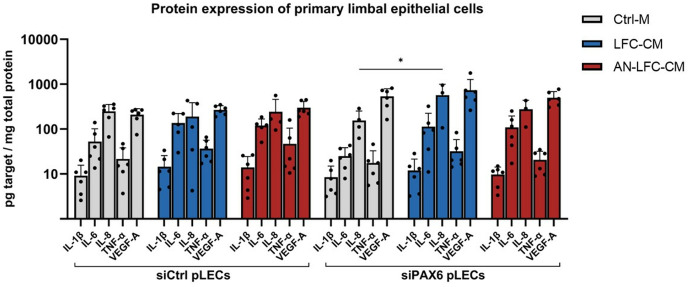
Protein level of interleukins (IL-1β, IL-6, IL-8), tumor necrosis factor-α (TNF-α) and vascular endothelial growth factor A (VEGF-A) in primary limbal epithelial cells transfected with a non-specific control siRNA (siCtrl pLECs) and limbal epithelial cells transfected with siRNA targeting PAX6 (siPAX6 pLECs) after treatment with control medium (Ctrl-M), conditioned medium from healthy limbal fibroblasts (LFC-CM) or conditioned medium from aniridia limbal fibroblasts (AN-LFC-CM), using ELISA. The measured concentrations of the protein of interest in the cell culture supernatant were divided by the total protein concentration of the cell lysates in order to obtain the respective concentration in picogram per milligram of total protein. Data is displayed as mean ± standard deviation. * p < 0.05.

**Fig 4:**
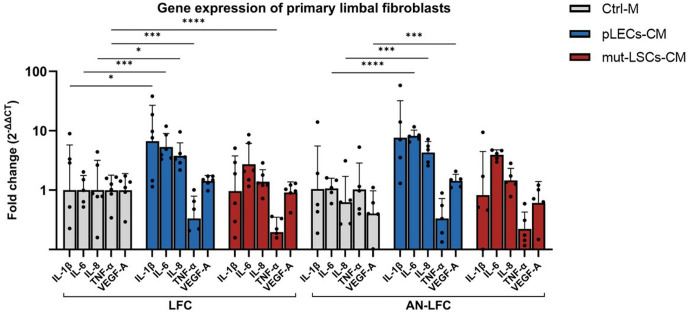
mRNA level of interleukins (IL-1β, IL-6, IL-8), tumor necrosis factor-α (TNF-α) and vascular endothelial growth factor A (VEGF-A) in healthy limbal fibroblasts (LFC) and aniridia limbal fibroblasts (AN-LFC) after treatment with conditioned medium of healthy primary limbal epithelial cells (pLECs-CM) or conditioned medium of an aniridia epithelial cell line (mut-LSCs-CM). Fold changes are expressed in relation to the LFC with control medium (Ctrl-M) as geometric mean ± geometric standard deviation. * p < 0.05; *** p < 0.001; **** p < 0.0001.

**Fig 5:**
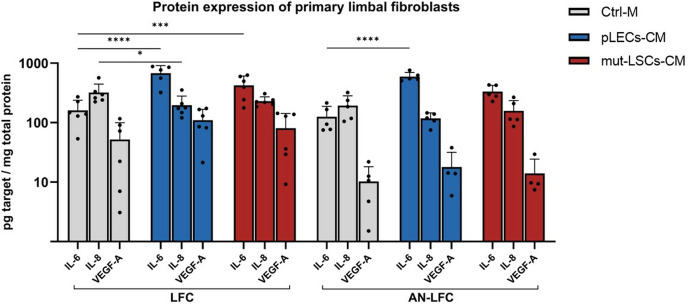
Protein level of interleukins (IL-6, IL-8) and vascular endothelial growth factor A (VEGF-A) in healthy limbal fibroblasts (LFC) and aniridia limbal fibroblasts (AN-LFC) after treatment with control medium (Ctrl-M), conditioned medium of healthy primary limbal epithelial cells (pLECs-CM) or conditioned medium of an aniridia epithelial cell line (mut-LSCs-CM). The measured concentrations of the protein of interest in the cell culture supernatant were divided by the total protein concentration of the cell lysates in order to obtain the respective concentration in picogram per milligram of total protein. The protein concentration levels of IL-1β and TNF-α in the cell culture supernatant were below the detection limit. Data is displayed as mean ± standard deviation. * p < 0.05; *** p < 0.001; **** p < 0.0001.

## Results

### PAX6 knockdown

To establish a model mimicking PAX6 haploinsufficiency, pLECs were transfected with siRNA targeting PAX6. Successful siRNA-mediated knockdown of the PAX6 protein was confirmed by western blot analysis, as shown in Fig 1.

### Gene and protein expression of primary limbal epithelial cells

The treatment of primary limbal epithelial cells (siCtrl pLECs or siPAX6 pLECs) with either conditioned medium from primary limbal fibroblasts (LFC-CM or AN-LFC-CM) revealed no statistically significant changes in the messenger RNA (mRNA) expression levels of the proinflammatory cytokines IL-1β, IL-6, IL-8, TNF-α and VEGF-A, as provided in Fig 2 and S3 Table.

The findings at the transcriptional level were largely mirrored in the protein expression data. Notably, only siPAX6 pLECs exhibited a significant increase of IL-8 protein levels in the cell culture supernatant after incubation with LFC-CM (p = 0.02). In contrast, there were no other statistically significant changes in the expression levels of IL-1β, IL-6, TNF-α and VEGF-A in the siCtrl pLECs or siPAX6 pLECs across all treatment groups, as shown in Fig 3 and listed in S4 Table.

Taken together these results suggest that the secretome of limbal fibroblasts minimally inflames the limbal epithelial cells.

### Gene and protein expression of primary limbal fibroblasts

Exposure of LFC to the pLECs-CM resulted in a significant upregulation of the mRNA expression of IL-1β (p = 0.03), IL-6 (p = 0.002) and IL-8 (p = 0.02), while TNF-α mRNA expression was significantly downregulated (p = 0.003). Similarly, the AN-LFC responded to the pLECs-CM with a significant upregulation of mRNA expression of IL-6 (p < 0.0001), IL-8 (p = 0.001) and VEGF-A (p = 0.001).

However, the treatment with mut-LSCs-CM resulted only in a significant downregulation of TNF-α mRNA expression in the LFC (p = 0.0001), while the expression levels of all other investigated cytokines remained unchanged in both LFC and AN-LFC. The effects of epithelial cell-conditioned media on mRNA expression in primary limbal fibroblasts are summarized in Fig 4 and presented in detail in S5 Table.

At the protein level, treatment with pLECs-CM led to a highly significant increase in IL-6 concentration in the cell culture supernatants of both the LFC and AN-LFC (p < 0.0001). In contrast, the incubation with mut-LSCs-CM caused only a significant increase in the protein expression of IL-6 in the LFC (p = 0.006) but not in the AN-LFC. Furthermore, the LFC with pLECs-CM demonstrated a significant decrease in IL-8 protein expression (p = 0.03). IL-1β and TNF-α protein were not detectable in the cell culture supernatants of LFC or AN-LFC. The results of the protein expression analysis for the limbal fibroblasts are displayed in Fig 5 and S6 Table.

The expression changes of the limbal fibroblasts depending on the type of epithelial cell conditioned medium hint towards functional differences in the secretome of healthy epithelial cells compared to the aniridia epithelial cell line.

## Discussion

The significance of limbal fibroblasts for the proliferation, differentiation and survival of limbal epithelial cells has been confirmed in numerous experimental studies [25–27]. In a mouse model, Amirjamshidi et al. demonstrated that corneas treated with CM from limbal fibroblasts after full limbus-to-limbus epithelial debridement exhibited significantly higher rates of corneal epithelial cell proliferation and reduced conjunctival overgrowth of the corneal surface, highlighting the role of limbal fibroblasts in supporting corneal epithelial integrity [26]. Furthermore, in a rabbit model, Espana et al. observed that the limbal stroma sustains the undifferentiated state of limbal epithelial progenitor cells with less apoptosis, while differentiation of epithelial cells is induced by the corneal stroma once they leave the limbal stem cell niche [27].

In this study, the gene and protein expression of the analyzed inflammatory biomarkers remained largely unchanged for both the siCtrl pLECs and siPAX6 pLECs treated with LFC-CM or AN-LFC-CM, with the exception of a significant increase of IL-8 in the cell culture supernatant of the siPAX6 pLECs that had been exposed to the LFC-CM. The secreted molecules by the limbal fibroblasts under the conditions studied appear to be insufficient to induce a severe inflammatory response in limbal epithelial cells. This finding aligns with the prevailing consensus, that limbal fibroblasts as stromal niche cells serve a supportive role, rather than to instigate inflammatory processes within limbal epithelial cells [28]. This perspective is further supported by Garfias et al., who demonstrated the immunosuppressive properties of limbal mesenchymal stem cells, particularly through the secretion of soluble factors such as Transforming Growth Factor-β1, which can suppress T-cell proliferation [29]. Conversely, the expression of IL-1β, IL-6, IL-8, TNF-α and VEGF-A may follow a distinct time pattern that was not captured at the selected time point in this study, emphasizing the need for analyses across multiple time intervals in future studies.

Moreover, it is noteworthy that, apart from the significant increase in IL-8 protein expression in the cell culture supernatant of siPAX6 pLECs after treatment with LFC-CM, no other discernible expression changes were observed when comparing the two CM. This raises the question of how PAX6 haploinsufficiency in congenital aniridia affects the unique secretome of limbal fibroblasts, especially given that mesenchymal-derived cells typically express minimal to no PAX6 [3].

In LFC, the treatment with pLECs-CM has been demonstrated to result in a significant increase in gene expression of IL-1β, IL-6 and IL-8, accompanied by a significant increase in protein expression of IL-6. In contrast, the LFC treated with mut-LSCs-CM only showed notable elevation in IL-6 protein expression. In the AN-LFC, the treatment with pLECs-CM led to a significant increase in IL-6, IL-8 and VEGF-A mRNA expression and in IL-6 protein expression, whereas the mut-LSCs-CM had no significant impact on gene or protein expression of the studied inflammatory biomarkers. In this study, the administration of pLECs-CM appears to elicit a more pronounced inflammatory response measured by the increase of cytokine expression in the limbal fibroblasts compared to the mut-LSCs-CM. This points to functional differences in the secretome of healthy limbal epithelial cells and the aniridia epithelial cell line. It is also conceivable that the upregulation of inflammatory cytokines in limbal fibroblasts following exposure to the pLECs-CM reflects a mechanism by which the pLECs regulate the limbal fibroblasts as their niche cells, thereby maintaining balance within the limbal stem cell niche. This hypothesis is supported by the *in vitro* observations of Bondarenko et al., who noted a reduction in proliferation and impaired migration of limbal stromal cells following treatment with CM from LESC [30]. Furthermore, Veréb et al. demonstrated that human LESC compared to differentiated corneal epithelial cells express higher levels of several immune-related genes, including IL-1β, IL-1α, IL-6, IL-8 and VEGF-A [31]. In contrast, the failure of mut-LSCs-CM to induce a similar inflammatory response in limbal fibroblasts may represent a breakdown in this regulatory feedback loop, which could contribute to progressive niche destabilization as seen in AAK [15]. Taken together, these findings support the idea that LESC actively contribute to the immune microenvironment, potentially influencing both epithelial and stromal cell behavior.

Furthermore, the LFC showed a significant decrease in TNF-α mRNA expression after incubation with pLECs-CM or mut-LSCs-CM, which was not found in the AN-LFC. This may suggest the existence of a potential self-regulatory feedback loop in LFC, that serves to prevent an excessive inflammatory response through downregulation of TNF-α, a key mediator of inflammatory processes [32].

Although the specific factors secreted by limbal fibroblasts that promote stemness in limbal epithelial cells are not fully elucidated, IL-6 emerges as a promising candidate for further investigations [25,28]. IL-6 is a versatile cytokine with a dual role in inflammation, both promoting and inhibiting inflammatory processes, and is involved in various physiological functions such as corneal wound healing, where it has been shown to facilitate the corneal epithelial cell migration [33,34]. Notara et al. observed that human limbal epithelial cells expressed elevated levels of putative stem cell markers and demonstrated the ability to form holoclones in an *in vitro* coculture with human limbal fibroblasts at a 3:1 ratio. The proliferative capacity as measured by the colony forming efficiency was found to be significantly reduced after inhibition of signal transducer and activator 3 (STAT3) and IL-6, which led Notara et al. to conclude that IL-6 may be critical for preserving a progenitor state of limbal epithelial cells through a STAT3-mediated pathway [25]. In this study, the gene and protein expression of IL-6 was significantly upregulated in the LFC group treated with pLECs-CM, whereas the mut-LSCs-CM failed to significantly increase IL-6 gene or protein expression in the respective AN-LFC group. This result implies that the mut-LSCs-CM may contain lower levels of the essential factors for sustaining IL-6 expression in AN-LFC during cellular interaction or that AN-LFC are less sensitive to these factors. However, it should be highlighted that the interaction between primary limbal epithelial cells and limbal fibroblasts was assessed in a one-way manner, using conditioned medium without direct co-culture. As such, the study lacks the various cell communication mechanisms present in the microenvironment of the limbal stem cell niche, which include direct interactions with several cell types like melanocytes, as well as extracellular matrix components and biomechanical forces that are crucial for regulating cytokine and growth factor expression [35–37].

In conclusion, limbal fibroblasts appear to fulfil a supportive role within the limbal stem cell niche as their secretome fails to induce a severe inflammatory response in limbal epithelial cells. The treatment of limbal fibroblasts with pLECs-CM, as opposed to mut-LSCs-CM, resulted in significant expression changes of the majority of the studied inflammatory biomarkers, potentially indicating a regulatory mechanism of healthy limbal epithelial cells aimed at preserving balance within the stem cell niche and emphasizes the importance of a controlled interaction. Unlike the healthy counterpart, within their respective AN-LFC group the mut-LSCs-CM did not significantly increase the gene or protein expression of IL-6, a putative cytokine that promotes stemness, suggesting an aberrant interaction in congenital aniridia. Further research into the specific factors secreted by limbal epithelial cells and limbal fibroblasts, their expression dynamics and regulatory mechanisms is required in order to develop effective therapies to manage ocular inflammation and preserve the limbal stem cell niche.

## Supporting information

S1 Table Descriptive data of donors. Descriptive data of donors including age, gender and stage of aniridia associated keratopathy; with n/a indicating unavailable information.
(DOCX)

S2 Table qPCR primers, ELISA kits and western blot antibody. Primers used for quantitative polymerase chain reaction (qPCR), kits used for enzyme-linked immunosorbent assay (ELISA) and antibody used for western blot analysis.
(DOCX)

S3 Table Gene expression of primary limbal epithelial cells. mRNA level of interleukins (IL-1β, IL-6, IL-8), tumor necrosis factor-α (TNF-α) and vascular endothelial growth factor A (VEGF-A) in primary limbal epithelial cells transfected with a non-specific control siRNA (siCtrl pLECs) and limbal epithelial cells transfected with siRNA targeting PAX6 (siPAX6 pLECs) after treatment with conditioned medium from healthy limbal fibroblasts (LFC-CM) or conditioned medium from aniridia limbal fibroblasts (AN-LFC-CM). Fold changes are expressed in relation to the siCtrl pLECs with control medium (Ctrl-M) as geometric mean ± geometric standard deviation. Respective p-values are provided in round brackets, followed by the number of replicates in square brackets.
(DOCX)

S4 Table Protein expression of primary limbal epithelial cells. Protein level of interleukins (IL-1β, IL-6, IL-8), tumor necrosis factor-α (TNF-α) and vascular endothelial growth factor A (VEGF-A) in primary limbal epithelial cells transfected with a non-specific control siRNA (siCtrl pLECs) and limbal epithelial cells transfected with siRNA targeting PAX6 (siPAX6 pLECs) after treatment with control medium (Ctrl-M), conditioned medium from healthy limbal fibroblasts (LFC-CM) or conditioned medium from aniridia limbal fibroblasts (AN-LFC-CM). The measured concentrations of the protein of interest in the cell culture supernatant were divided by the total protein concentration of the cell lysates in order to obtain the respective concentration in picogram per milligram of total protein. Data is displayed as mean ± standard deviation. Respective p-values are provided in round brackets, followed by the number of replicates in square brackets. Significant p-values <0.05 were highlighted in bold font.
(DOCX)

S5 Table Gene expression of primary limbal fibroblasts. mRNA level of interleukins (IL-1β, IL-6, IL-8), tumor necrosis factor-α (TNF-α) and vascular endothelial growth factor A (VEGF-A) in healthy limbal fibroblasts (LFC) and aniridia limbal fibroblasts (AN-LFC) after treatment with conditioned medium of healthy primary limbal epithelial cells (pLECs-CM) or conditioned medium of an aniridia epithelial cell line (mut-LSCs-CM). Fold changes are expressed in relation to the LFC with control medium (Ctrl-M) as geometric mean ± geometric standard deviation. Respective p-values are provided in round brackets, followed by the number of replicates in square brackets. Significant p-values <0.05 were highlighted in bold font.
(DOCX)

S6 Table Protein expression of primary limbal fibroblasts. Protein level of interleukins (IL-1β, IL-6, IL-8), tumor necrosis factor-α (TNF-α) and vascular endothelial growth factor A (VEGF-A) in healthy limbal fibroblasts (LFC) and aniridia limbal fibroblasts (AN-LFC) after treatment with control medium (Ctrl-M), conditioned medium of healthy primary limbal epithelial cells (pLECs-CM) or conditioned medium of an aniridia epithelial cell line (mut-LSCs-CM). The measured concentrations of the protein of interest in the cell culture supernatant were divided by the total protein concentration of the cell lysates in order to obtain the respective concentration in picogram per milligram of total protein. The protein concentration levels of IL-1β and TNF-α in the cell culture supernatant were below the detection limit. Data is displayed as mean ± standard deviation. Respective p-values are provided in round brackets, followed by the number of replicates in square brackets. Significant p-values <0.05 were highlighted in bold font.
(DOCX)

S1 Fig Schematic illustration of the limbal epithelial stem cell niche. LESC are presumably distributed in clusters within the basal epithelial layers of the crypts situated between the vascularized Vogt´s palisades [10–12].
(TIF)

S2 Fig Protocol figure. Healthy primary limbal epithelial cells (pLECs) and healthy (LFC) or aniridia primary limbal fibroblasts (AN-LFC) were isolated. A PAX6-deficient limbal epithelial cell line (mut-LSCs) modeled aniridia. pLECs underwent siRNA-mediated PAX6 knockdown (siPAX6 pLECs) with control cells transfected with non-specific siRNA (siCtrl pLECs). siCtrl and siPAX6 pLECs were treated with LFC-CM and AN-LFC-CM for 24 hours, while LFC and AN-LFC were treated with pLECs-CM and mut-LSCs-CM for 48 hours. Successful siRNA-mediated knockdown of the PAX6 protein was confirmed by western blot analysis. Gene and protein expression of IL-1β, IL-6, IL-8, TNF-α and VEGF-A were measured using qPCR and ELISA.
(TIF)
