# Supplementary material for: Reciprocal effects of conditioned medium on gene and protein expression of limbal epithelial cells and limbal fibroblasts in congenital aniridia
Source: PLoS One. 2025 Jul 7;20(7):e0327167. doi: 10.1371/journal.pone.0327167 (PMC12233234; doi:10.1371/journal.pone.0327167)
Supplement: S1 Table — Descriptive data of donors including age, gender and stage of aniridia associated keratopathy; with n/a indicating unavailable information. (DOCX) [file pone.0327167.s001.docx]

**S1 Table. Descriptive data of donors.** Descriptive data of donors including age, gender and stage of aniridia associated keratopathy; with n/a indicating unavailable information.

|  |  | **Age** | **Gender** | **AAK stage** |
| --- | --- | --- | --- | --- |
| **Cells used for treatment** | **pLECs** | n/a | n/a | - |
|  |  | 75 | f | - |
|  |  | 72 | m | - |
|  |  | 65 | f | - |
|  |  | 54 | m | - |
|  |  | 75 | f | - |
|  |  | 81 | f | - |
|  |  | 63 | f | - |
|  | **LFC** | 76 | m | - |
|  |  | 85 | m | - |
|  |  | 98 | f | - |
|  |  | 65 | m | - |
|  |  | 76 | m | - |
|  |  | 78 | m | - |
|  | **AN-LFC** | 16 | f | 4 |
|  |  | 2 | f | n/a |
|  |  | 30 | m | 3 |
|  |  | 59 | f | 4 |
|  |  | 78 | m | 4 |
| **Cells used for collection of conditioned medium** | **pLECs** | 81 | f | - |
|  |  | 74 | m | - |
|  |  | 76 | f | - |
|  |  | 70 | m | - |
|  |  | n/a | n/a | - |
|  |  | n/a | n/a | - |
|  |  | 62 | m | - |
|  |  | 82 | n/a | - |
|  |  | 52 | m | - |
|  |  | 91 | f | - |
|  |  | 70 | n/a | - |
|  |  | 72 | f | - |
|  |  | 87 | m | - |
|  |  | 84 | m | - |
|  |  | 64 | m | - |
|  |  | 55 | m | - |
|  | **LFC** | 76 | m | - |
|  |  | 85 | m | - |
|  |  | 83 | f | - |
|  |  | 98 | f | - |
|  |  | 77 | m | - |
|  |  | 76 | m | - |
|  |  | 78 | m | - |
|  | **AN-LFC** | 50 | f | 4 |
|  |  | 16 | f | 4 |
|  |  | 2 | f | n/a |
|  |  | 64 | f | 4 |
|  |  | 30 | m | 3 |
|  |  | 46 | f | 5 |
|  |  | 59 | f | 4 |
