# Supplementary material for: Reciprocal effects of conditioned medium on gene and protein expression of limbal epithelial cells and limbal fibroblasts in congenital aniridia
Source: PLoS One. 2025 Jul 7;20(7):e0327167. doi: 10.1371/journal.pone.0327167 (PMC12233234; doi:10.1371/journal.pone.0327167)
Supplement: S2 Table — Primers used for quantitative polymerase chain reaction (qPCR), kits used for enzyme-linked immunosorbent assay (ELISA) and antibody used for western blot analysis. (DOCX) [file pone.0327167.s002.docx]

**S2 Table. qPCR primers, ELISA kits and western blot antibody.** Primers used for quantitative polymerase chain reaction (qPCR), kits used for enzyme-linked immunosorbent assay (ELISA) and antibody used for western blot analysis.

| **Primer** | **Amplicon length (bp)** | **Catalog number** | **Manufacturer** |
| --- | --- | --- | --- |
| GUSB | 96 | QT00046046 | QIAGEN N.V., Venlo, The Netherlands |
| IL-1β | 117 | QT00021385 |  |
| IL-6 | 107 | QT00083720 |  |
| IL-8 | 102 | QT00000322 |  |
| TBP | 132 | QT00000721 |  |
| TNF-α | 98 | QT00029162 |  |
| VEGF-A | 150, 204, 222, 273 | QT01010184 |  |
| **ELISA kit** |  |  |  |
| IL-1β | - | DY201 | R&D Systems Inc., Minneapolis, MN, USA |
| IL-6 | - | DY206 |  |
| IL-8 | - | DY208 |  |
| TNF-α | - | DY210 |  |
| VEGF-A | - | DY293B |  |
| **Antibody** |  |  |  |
| PAX6 | - | AB2237 | Merck KGaA, Darmstadt, Germany |
