# Supplementary material for: Reciprocal effects of conditioned medium on gene and protein expression of limbal epithelial cells and limbal fibroblasts in congenital aniridia
Source: PLoS One. 2025 Jul 7;20(7):e0327167. doi: 10.1371/journal.pone.0327167 (PMC12233234; doi:10.1371/journal.pone.0327167)
Supplement: S3 Table — mRNA level of interleukins (IL-1β, IL-6, IL-8), tumor necrosis factor-α (TNF-α) and vascular endothelial growth factor A (VEGF-A) in primary limbal epithelial cells transfected with a non-specific control siRNA (siCtrl pLECs) and limbal epithelial cells transfected with siRNA targeting PAX6 (siPAX6 pLECs) after treatment with conditioned medium from healthy limbal fibroblasts (LFC-CM) or conditioned medium from aniridia limbal fibroblasts (AN-LFC-CM). Fold changes are expressed in relation to the siCtrl pLECs with control medium (Ctrl-M) as geometric mean ± geometric standard deviation. Respective p-values are provided in round brackets, followed by the number of replicates in square brackets. (DOCX) [file pone.0327167.s003.docx]

**S3 Table. Gene expression of primary limbal epithelial cells.** mRNA level of interleukins (IL-1β, IL-6, IL-8), tumor necrosis factor-α (TNF-α) and vascular endothelial growth factor A (VEGF-A) in primary limbal epithelial cells transfected with a non-specific control siRNA (siCtrl pLECs) and limbal epithelial cells transfected with siRNA targeting PAX6 (siPAX6 pLECs) after treatment with conditioned medium from healthy limbal fibroblasts (LFC-CM) or conditioned medium from aniridia limbal fibroblasts (AN-LFC-CM). Fold changes are expressed in relation to the siCtrl pLECs with control medium (Ctrl-M) as geometric mean ± geometric standard deviation. Respective p-values are provided in round brackets, followed by the number of replicates in square brackets.

| **Gene** | **Limbal epithelial cells - fold changes (2^-ΔΔCT^), p-values and replicates** | | | | | |
| --- | --- | --- | --- | --- | --- | --- |
|  | **siCtrl pLECs** | | | **siPAX6 pLECs** | | |
|  | **Ctrl-M** | **LFC-CM** | **AN-LFC-CM** | **Ctrl-M** | **LFC-CM** | **AN-LFC-CM** |
| IL-1β | 1.0 [5] | 1.25 ± 3.19 (0.40) [6] | 2.04 ± 2.13 (0.16) [6] | 0.60 ± 2.26 [6] | 0.84 ± 2.36 (0.87) [6] | 0.93 ± 2.19 (0.84) [6] |
| IL-6 | 1.0 [5] | 1.80 ± 2.53 (0.37) [5] | 2.28 ± 2.57 (0.09) [5] | 0.37 ± 3.41 [5] | 0.49 ± 3.11 (0.97) [5] | 0.55 ± 2.86 (0.96) [5] |
| IL-8 | 1.0 [6] | 0.81 ± 1.67 (0.42) [6] | 0.78 ± 1.74 (0.35) [6] | 0.42 ± 1.51 [6] | 0.40 ± 1.43 (0.99) [6] | 0.40 ± 1.53 (0.99) [6] |
| TNF-α | 1.0 [5] | 0.76 ± 1.30 (0.54) [5] | 0.94 ± 1.38 (0.98) [5] | 0.35 ± 2.04 [5] | 0.37 ± 2.50 (0.87) [5] | 0.48 ± 2.10 (0.71) [5] |
| VEGF-A | 1.0 [6] | 1.25 ± 1.21 (0.55) [6] | 1.09 ± 1.30 (0.90) [6] | 1.44 ± 1.40 [6] | 1.55 ± 1.50 (0.72) [6] | 1.80 ± 1.39 (0.28) [6] |
