# Supplementary material for: Reciprocal effects of conditioned medium on gene and protein expression of limbal epithelial cells and limbal fibroblasts in congenital aniridia
Source: PLoS One. 2025 Jul 7;20(7):e0327167. doi: 10.1371/journal.pone.0327167 (PMC12233234; doi:10.1371/journal.pone.0327167)
Supplement: S4 Table — Protein level of interleukins (IL-1β, IL-6, IL-8), tumor necrosis factor-α (TNF-α) and vascular endothelial growth factor A (VEGF-A) in primary limbal epithelial cells transfected with a non-specific control siRNA (siCtrl pLECs) and limbal epithelial cells transfected with siRNA targeting PAX6 (siPAX6 pLECs) after treatment with control medium (Ctrl-M), conditioned medium from healthy limbal fibroblasts (LFC-CM) or conditioned medium from aniridia limbal fibroblasts (AN-LFC-CM). The measured concentrations of the protein of interest in the cell culture supernatant were divided by the total protein concentration of the cell lysates in order to obtain the respective concentration in picogram per milligram of total protein. Data is displayed as mean ± standard deviation. Respective p-values are provided in round brackets, followed by the number of replicates in square brackets. Significant p-values <0.05 were highlighted in bold font. (DOCX) [file pone.0327167.s004.docx]

**S4 Table. Protein expression of primary limbal epithelial cells.** Protein level of interleukins (IL-1β, IL-6, IL-8), tumor necrosis factor-α (TNF-α) and vascular endothelial growth factor A (VEGF-A) in primary limbal epithelial cells transfected with a non-specific control siRNA (siCtrl pLECs) and limbal epithelial cells transfected with siRNA targeting PAX6 (siPAX6 pLECs) after treatment with control medium (Ctrl-M), conditioned medium from healthy limbal fibroblasts (LFC-CM) or conditioned medium from aniridia limbal fibroblasts (AN-LFC-CM). The measured concentrations of the protein of interest in the cell culture supernatant were divided by the total protein concentration of the cell lysates in order to obtain the respective concentration in picogram per milligram of total protein. Data is displayed as mean ± standard deviation. Respective p-values are provided in round brackets, followed by the number of replicates in square brackets. Significant p-values <0.05 were highlighted in bold font.

| **Protein** | **Limbal epithelial cells – protein expression (pg target / mg of total protein), p-values and replicates** | | | | | |
| --- | --- | --- | --- | --- | --- | --- |
|  | **siCtrl pLECs** | | | **siPAX6 pLECs** | | |
|  | **Ctrl-M** | **LFC-CM** | **AN-LFC-CM** | **Ctrl-M** | **LFC-CM** | **AN-LFC-CM** |
| IL-1β | 9.16 ± 6.47 [6] | 14.53 ± 11.01 (0.44) [6] | 13.96 ± 10.45 (0.52) [6] | 8.50 ± 6.49 [6] | 11.99 ± 9.41 (0.70) [6] | 9.71 ± 4.52 (0.96) [6] |
| IL-6 | 52.85 ± 48.08 [6] | 136.46 ± 84.14 (0.13) [5] | 119.35 ± 48.16 (0.25) [5] | 25.11 ± 13.38 [6] | 113.70 ± 111.49 (0.08) [6] | 109.22 ± 86.94 (0.10) [6] |
| IL-8 | 247.43 ± 106.70 [6] | 189.92 ± 196.98 (0.85) [5] | 135.89 ± 37.32 (0.64) [4] | 155.67 ± 96.64 [4] | 567.82 ± 428.50 (**0.02**) [3] | 277.24 ± 161.32 (0.63) [3] |
| TNF-α | 21.70 ± 16.56 [6] | 36.62 ± 20.03 (0.58) [6] | 47.13 ± 58.21 (0.24) [6] | 17.56 ± 15.07 [6] | 32.04 ± 26.33 (0.60) [6] | 20.68 ± 11.35 (0.98) [6] |
| VEGF-A | 209.84 ± 75.32 [6] | 268.89 ± 62.54 (0.89) [6] | 299.42 ± 112.83 (0.78) [6] | 536.72 ± 252.55 [6] | 732.24 ± 536.60 (0.34) [6] | 500.70 ± 180.89 (0.96) [6] |
