# Supplementary material for: Reciprocal effects of conditioned medium on gene and protein expression of limbal epithelial cells and limbal fibroblasts in congenital aniridia
Source: PLoS One. 2025 Jul 7;20(7):e0327167. doi: 10.1371/journal.pone.0327167 (PMC12233234; doi:10.1371/journal.pone.0327167)
Supplement: S5 Table — mRNA level of interleukins (IL-1β, IL-6, IL-8), tumor necrosis factor-α (TNF-α) and vascular endothelial growth factor A (VEGF-A) in healthy limbal fibroblasts (LFC) and aniridia limbal fibroblasts (AN-LFC) after treatment with conditioned medium of healthy primary limbal epithelial cells (pLECs-CM) or conditioned medium of an aniridia epithelial cell line (mut-LSCs-CM). Fold changes are expressed in relation to the LFC with control medium (Ctrl-M) as geometric mean ± geometric standard deviation. Respective p-values are provided in round brackets, followed by the number of replicates in square brackets. Significant p-values <0.05 were highlighted in bold font. (DOCX) [file pone.0327167.s005.docx]

**S5 Table. Gene expression of primary limbal fibroblasts.** mRNA level of interleukins (IL-1β, IL-6, IL-8), tumor necrosis factor-α (TNF-α) and vascular endothelial growth factor A (VEGF-A) in healthy limbal fibroblasts (LFC) and aniridia limbal fibroblasts (AN-LFC) after treatment with conditioned medium of healthy primary limbal epithelial cells (pLECs-CM) or conditioned medium of an aniridia epithelial cell line (mut-LSCs-CM). Fold changes are expressed in relation to the LFC with control medium (Ctrl-M) as geometric mean ± geometric standard deviation. Respective p-values are provided in round brackets, followed by the number of replicates in square brackets. Significant p-values <0.05 were highlighted in bold font.

| **Gene** | **Limbal fibroblasts - fold changes (2^-ΔΔCT^), p-values and replicates** | | | | | |
| --- | --- | --- | --- | --- | --- | --- |
|  | **LFC** | | | **AN-LFC** | | |
|  | **Ctrl-M** | **pLECs-CM** | **mut-LSCs-CM** | **Ctrl-M** | **pLECs-CM** | **mut-LSCs-CM** |
| IL-1β | 1.0 [6] | 6.66 ± 4.03 (**0.03**) [6] | 0.96 ± 3.93 (0.97) [6] | 0.55 ± 2.61 [5] | 4.62 ± 2.76 (0.41) [5] | 0.45 ± 3.19 (0.99) [5] |
| IL-6 | 1.0 [5] | 5.31 ± 1.69 (**0.002**) [6] | 2.73 ± 2.24 (0.13) [6] | 1.07 ± 1.49 [5] | 8.21 ± 1.25 (**<0.0001**) [5] | 3.92 ± 1.22 (0.10) [5] |
| IL-8 | 1.0 [6] | 3.79 ± 1.65 (**0.02**) [6] | 1.39 ± 1.59 (0.99) [6] | 0.42 ± 1.66 [5] | 4.29 ± 1.54 (**0.001**) [5] | 1.46 ± 1.60 (0.48) [5] |
| TNF-α | 1.0 [6] | 0.33 ± 2.37 (**0.003**) [6] | 0.20 ± 1.77 (**0.0001**) [6] | 0.68 ± 1.68 [5] | 0.33 ± 2.16 (0.24) [5] | 0.22 ± 1.93 (0.06) [5] |
| VEGF-A | 1.0 [6] | 1.43 ± 1.24 (0.28) [6] | 0.92 ± 1.51 (0.67) [6] | 0.41 ± 2.41 [5] | 1.43 ± 1.29 (**0.001**) [5] | 0.61 ± 2.30 (0.57) [5] |
