# Supplementary material for: Reciprocal effects of conditioned medium on gene and protein expression of limbal epithelial cells and limbal fibroblasts in congenital aniridia
Source: PLoS One. 2025 Jul 7;20(7):e0327167. doi: 10.1371/journal.pone.0327167 (PMC12233234; doi:10.1371/journal.pone.0327167)
Supplement: S6 Table — Protein level of interleukins (IL-1β, IL-6, IL-8), tumor necrosis factor-α (TNF-α) and vascular endothelial growth factor A (VEGF-A) in healthy limbal fibroblasts (LFC) and aniridia limbal fibroblasts (AN-LFC) after treatment with control medium (Ctrl-M), conditioned medium of healthy primary limbal epithelial cells (pLECs-CM) or conditioned medium of an aniridia epithelial cell line (mut-LSCs-CM). The measured concentrations of the protein of interest in the cell culture supernatant were divided by the total protein concentration of the cell lysates in order to obtain the respective concentration in picogram per milligram of total protein. The protein concentration levels of IL-1β and TNF-α in the cell culture supernatant were below the detection limit. Data is displayed as mean ± standard deviation. Respective p-values are provided in round brackets, followed by the number of replicates in square brackets. Significant p-values <0.05 were highlighted in bold font. (DOCX) [file pone.0327167.s006.docx]

**S6 Table. Protein expression of primary limbal fibroblasts.** Protein level of interleukins (IL-1β, IL-6, IL-8), tumor necrosis factor-α (TNF-α) and vascular endothelial growth factor A (VEGF-A) in healthy limbal fibroblasts (LFC) and aniridia limbal fibroblasts (AN-LFC) after treatment with control medium (Ctrl-M), conditioned medium of healthy primary limbal epithelial cells (pLECs-CM) or conditioned medium of an aniridia epithelial cell line (mut-LSCs-CM). The measured concentrations of the protein of interest in the cell culture supernatant were divided by the total protein concentration of the cell lysates in order to obtain the respective concentration in picogram per milligram of total protein. The protein concentration levels of IL-1β and TNF-α in the cell culture supernatant were below the detection limit. Data is displayed as mean ± standard deviation. Respective p-values are provided in round brackets, followed by the number of replicates in square brackets. Significant p-values <0.05 were highlighted in bold font.

| **Protein** | **Limbal fibroblasts – protein expression (pg target / mg of total protein), p-values and replicates** | | | | | |
| --- | --- | --- | --- | --- | --- | --- |
|  | **LFC** | | | **AN-LFC** | | |
|  | **Ctrl-M** | **pLECs-CM** | **mut-LSCs-CM** | **Ctrl-M** | **pLECs-CM** | **mut-LSCs-CM** |
| IL-1β | not detectable | | | | | |
| IL-6 | 160.92 ± 76.70 [6] | 676.86 ± 234.89 (**<0.0001**) [5] | 423.60 ± 185.46 (**0.006**) [6] | 125.40 ± 62.03 [5] | 593.95 ± 103.08 (**<0.0001**) [5] | 332.07 ± 91.36 (0.06) [5] |
| IL-8 | 319.90 ± 126.72 [6] | 196.93 ± 82.41 (**0.03**) [6] | 230.06 ± 41.91 (0.12) [6] | 193.32 ± 89.25 [5] | 117.57 ± 28.16 (0.26) [5] | 157.81 ± 76.64 (0.72) [5] |
| TNF-α | not detectable | | | | | |
| VEGF-A | 52.01 ± 47.75 [6] | 109.75 ± 57.99 (0.058) [6] | 80.66 ± 61.91 (0.43) [6] | 10.27 ± 7.87 [5] | 17.97 ± 13.79 (0.95) [4] | 13.98 ± 10.33 (0.99) [4] |
