# Supplementary figures and images for: Reciprocal effects of conditioned medium on gene and protein expression of limbal epithelial cells and limbal fibroblasts in congenital aniridia
Source: PLoS One. 2025 Jul 7;20(7):e0327167. doi: 10.1371/journal.pone.0327167 (PMC12233234; doi:10.1371/journal.pone.0327167)

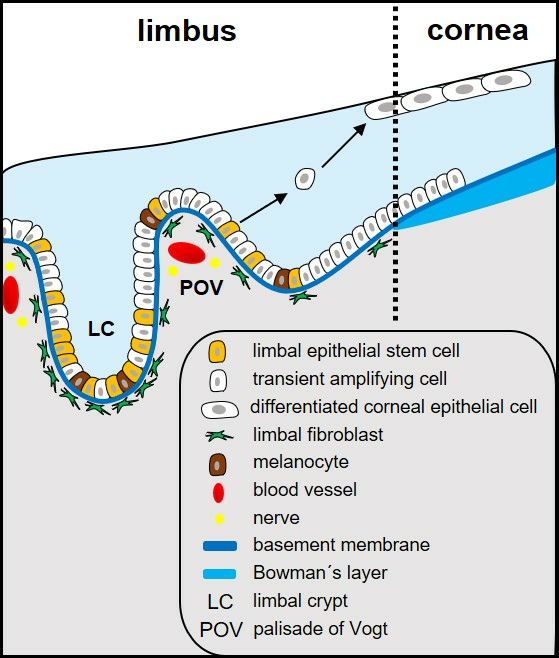

Supplement: S1 Fig — LESC are presumably distributed in clusters within the basal epithelial layers of the crypts situated between the vascularized Vogt´s palisades [10–12]. (TIF) [file pone.0327167.s007.tif]

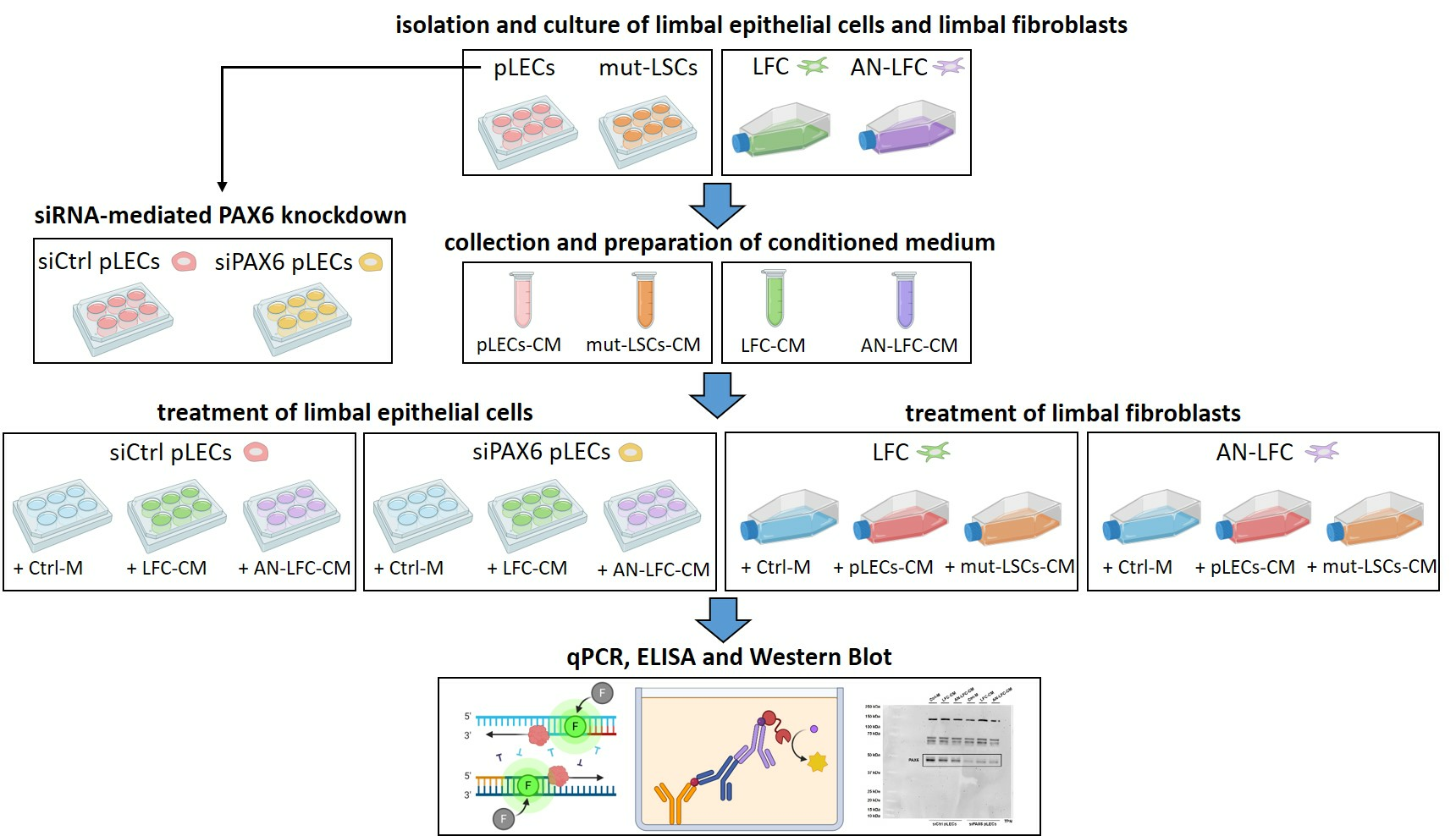

Supplement: S2 Fig — Healthy primary limbal epithelial cells (pLECs) and healthy (LFC) or aniridia primary limbal fibroblasts (AN-LFC) were isolated. A PAX6-deficient limbal epithelial cell line (mut-LSCs) modeled aniridia. pLECs underwent siRNA-mediated PAX6 knockdown (siPAX6 pLECs) with control cells transfected with non-specific siRNA (siCtrl pLECs). siCtrl and siPAX6 pLECs were treated with LFC-CM and AN-LFC-CM for 24 hours, while LFC and AN-LFC were treated with pLECs-CM and mut-LSCs-CM for 48 hours. Successful siRNA-mediated knockdown of the PAX6 protein was confirmed by western blot analysis. Gene and protein expression of IL-1β, IL-6, IL-8, TNF-α and VEGF-A were measured using qPCR and ELISA. (TIF) [file pone.0327167.s008.tif]
